# Hyponatremia in patients with systemic lupus erythematosus

**DOI:** 10.1038/srep25566

**Published:** 2016-05-19

**Authors:** Jae Il Shin, Se Jin Park, Chang-Hee Suh, Geum Hwa Lee, Min Woo Hur, Song Yi Han, Dong Soo Kim, Ji Hong Kim

**Affiliations:** 1 Department of Pediatrics, Yonsei University College of Medicine, Seoul, Korea; 2 Department of Pediatric Nephrology, Severance Children’s Hospital, Seoul, Korea; 3 Department of Pediatrics, Ajou University School of Medicine, Daewoo General Hospital, Geoje, Korea; 4 Department of Rheumatology, Ajou University School of Medicine, Suwon, Korea; 5 Department of Pediatrics, Gangnam Severance Hospital, Yonsei University College of Medicine, Seoul, Korea

## Abstract

The aim of this study was to determine whether decreased serum sodium concentration could be associated with the disease activity in SLE. We retrospectively analyzed the data of the two independent cohorts of children and adults with SLE in two centers. Hyponatremia was associated with serum chloride (p = 0.004), albumin (p = 0.002) and SLE disease activity index (SLEDAI) (p = 0.026) in children with SLE. Serum sodium levels were correlated negatively with ESR (p =0.001) and positively with serum albumin levels (p < 0.0001) and C3 (p = 0.008) in children with SLE and those levels were correlated negatively with serum interleukin-6 levels (p = 0.003) in adults with SLE. Independent risk factors for the development of hyponatremia were the decreased serum C3 levels (OR 1.069, p = 0.031), the decreased serum chloride levels (OR 2.054, p = 0.006) and increased erythrocyte sedimentation rate (ESR) (OR 1.066, p = 0.03) in children with SLE and increased C-reactive protein (CRP) (OR 1.480, p = 0.023) in combined cohorts with SLE by multiple logistic regression analyses. Our study firstly showed that hyponatremia could reflect a disease activity and severe inflammation of SLE.

Systemic lupus erythematosus (SLE) is a chronic multisystem autoimmune disease with a highly variable clinical course^1^,^2^. During the course of the disease, decreased serum levels of C3, C4 and high anti-double stranded (anti-ds) DNA antibody titers are reported to be associated with the disease activity and poor survival^3^.

Hyponatremia is known to be one of the most common electrolyte abnormalities and can cause seizures, brain edema, encephalopathy, and even death^4^,^5^. Hyponatremia has been reported to be poor clinical outcome such as mortality in general population, dialyzed patients and those with heart failure, cirrhosis and sepsis^6^,^7^,^8^. However, the incidence and clinical significance of hyponatremia has not been studied in rheumatic diseases including SLE and only some case reports of hyponatremia have been reported in SLE^9^,^10^. Also, there has been no report describing the relationship between the degree of hyponatremia and the disease activity in both childhood and adulthood SLE.

Therefore, the aim of this study was to evaluate the clinical and laboratory characteristics related to disease activity in children and adults with SLE and determine whether decreased serum sodium concentration could be associated with the disease activity in SLE.

## Results

### A cohort of children and adolescents with SLE

Laboratory characteristics of the patients with (Group I, n = 11) and without hyponatremia (Group II, n = 63) are shown in Table 1. There were no significant differences in white blood cell (WBC) counts, hemoglobin, hematocrit, platelet counts, erythrocyte sedimentation rate (ESR), C-reactive protein (CRP), serum potassium, total carbon dioxide (tCO_2_), blood urea nitrogen (BUN), creatinine, total protein, cholesterol, uric acid, alanine aminotransferase (ALT) and total bilirubin between the two groups (p > 0.05). However, serum chloride (p = 0.004), albumin (p = 0.002), aspartate aminotransferase (AST) levels (p = 0.004) and SLE disease activity index (SLEDAI) (p = 0.026) were significantly higher and serum calcium levels (p = 0.004) were significantly lower in SLE patients with hyponatremia than those without.

The positivity of antinuclear antibody (ANA), anti-ds DNA antibody and other autoantibodies, and the incidence of proteinuria did not differ between the two groups. However, serum C3 levels which reflect the disease activity were significantly lower in SLE patients with hyponatremia than those without (p = 0.004) (Table 2).

Independent risk factors for the development of hyponatremia were the decreased serum C3 levels (OR 1.069, p = 0.031), the decreased serum chloride levels (OR 2.054, p = 0.006) and increased erythrocyte sedimentation rate (ESR) (OR 1.066, p = 0.03) in children with SLE by multiple logistic regression analysis (Table 3).

The values of area under the curves (AUC) for the prediction of hyponatremia in children with SLE were 0.749 (95% CI 0.589–0.908, p = 0.026) for high SLEDAI, 0.779 (95% CI 0.613–0.946, p = 0.012) for high ESR levels, 0.775 (95% CI 0.599–0.951, p = 0.013) for high AST levels, 0.758 (95% CI 0.563–0.953, p = 0.021) for decreased serum C3 levels and 0.778 (95% CI 0.650–0.906, p = 0.013) for decreased serum albumin levels.

In addition, serum sodium levels were correlated negatively with ESR (r = −0.399, p = 0.001) and serum AST levels (r = −0.334, p = 0.004), but positively with C3 (r = 0.304, p = 0.008), serum chloride (r = 0.372, p = 0.001), serum calcium (r = 0.549, p < 0.0001) and serum albumin levels (r = 0.479, p < 0.0001) (Table 4).

Furthermore, changes in serum sodium levels (Na levels at the follow-up state– Na levels at the initial state, ΔNa) correlated positively with changes in serum C3 (ΔC3) (r = 0.476, p = 0.003), serum C4 (ΔC4) (r = 0.357, p = 0.03), hemoglobin (ΔHb) (r = 0.340, p = 0.039), hematocrit (ΔHct) (r = 0.425, p = 0.009), chloride (Δchloride) (r = 0.466, p = 0.004), calcium (Δcalcium) (r = 0.385, p = 0.019), phosphorus (Δphosphorus) (r = 0.391, p = 0.017) and albumin levels (Δalbumin) (r = 0.481, p = 0.003). However, ΔNa were not correlated with changes in SLEDAI (Δ SLEDAI) (r = −0.191, p = 0.313) (Table 5).

### A cohort of adolescents and adults with SLE

Laboratory characteristics of the patients with (n = 7) and without hyponatremia (n = 77) are shown in Supplementary Table S1. There were no significant differences in WBC counts, SLEDAI, interleukin (IL)-6, IL-2, the dose of steroids and urine specific gravity between the two groups (p > 0.05). CRP levels were higher in SLE patients with hyponatremia than those without with a borderline significance (p = 0.077).

Serum sodium levels were correlated negatively with serum IL-6 levels (r = −0.317, p = 0.003), while those did not correlate to serum IL-2 levels (p = 0.389). Serum IL-6 levels were also positively correlated with SLEDAI (r = 0.386, p = 0.032) (Table 6). The significant correlations between serum sodium and IL-6 levels were more prominent in SLE patients who were not treated with steroids (r = −0.424, p = 0.017) than those who were treated with steroids (r = −0.247, p = 0.075). In SLE patients who were not treated with steroids, serum sodium levels were correlated negatively with urine specific gravity which reflect the effect of ADH with a borderline significance (r = −0.326, p = 0.073) (Supplementary Tables S2–S3).

### Two combined cohorts of children and adults with SLE

In univariate analyses, there were no significant differences in WBC counts and SLEDAI between the two groups (p > 0.05) and ESR (p = 0.053) and CRP levels (p = 0.053) were higher in SLE patients with hyponatremia (n = 18) than those without (n = 140) with borderline significances (Supplementary Table S4).

Multiple logistic regression analysis showed that increased CRP level (OR 1.480, p = 0.023) was an independent risk factor for the development of hyponatremia in combined cohorts of children and adults with SLE (Supplementary Table S5). In addition, serum sodium levels were correlated negatively with CRP (r = −0.368, p < 0.0001) and ESR levels (r = −0.189, p = 0.023) (Supplementary Table S6).

### Receiver operating characteristic (ROC) curves

To establish the predictive value of the parameters for predicting hyponatremia in patients with lupus, ROC curves were plotted for SLEDAI, ESR, AST, serum C3 and albumin levels.

AUC values from ROC curve analyses for SLEDAI, ESR, AST, serum C3 and albumin levels were 0.743 (*P* = 0.029), 0.768 (*P* = 0.016), 0.770 (*P* = 0.015), 0.746 (*P* = 0.027) and 0.777 (*P* = 0.013), respectively (Supplementary Table S7 and Figure S1).

## Discussion

The main goal of this study was to investigate the significance of hyponatremia in children and adults with SLE and determine whether hyponatremia is associated with disease activity. Our study firstly demonstrated the association of hyponatremia with the decreased C3 and increased ESR levels and SLEDAI which reflect disease activity of SLE in a cohort of children with SLE and serum sodium levels were correlated negatively with serum IL-6 levels in an independent cohort of adults with SLE, which have not been investigated in both adults and children over the past decade.

Hyponatremia can occur in patients with SLE by various causes such as renal disease, acute kidney injury, and medication use, but only very few case reports were found in literatures on hyponatremia in SLE^9^,^10^. Although the exact mechanism of hyponatremia in SLE flare patients is not established yet, we speculate that inflammation can be one of the mechanisms in the pathogenesis of hyponatremia in SLE patients. In a cohort of children with SLE, we found that decreased serum chloride levels were independent risk factors for the development of hyponatremia in children with SLE. Urinary loss of sodium chloride could cause hyponatremia and hypochloremia and it can be caused by renal tubular damage or increased renal sodium loss by a reduction in renal tubular sodium absorption in inflammatory conditions^11^,^12^,^13^,^14^,^15^,^16^,^17^,^18^,^19^,^20^. However, we excluded the SLE patients with evident renal failure, because tubular injury can cause natriuresis and hyponatremia which obscure the relationship between lupus activity and serum sodium levels. Therefore, serum creatinine levels and the degree of proteinuria did not differ in SLE patients with hyponatremia than those without.

Eisenhut pointed out that inflammatory mediators such as IL-1 and tumor necrosis factor (TNF)-α have been reported to reduce sodium transport in epithelial cells by a reduction in the expression and function of the apical epithelial sodium channel (ENaC) and/or the sodium potassium ATPase (Na/K ATPase) at the basolateral membrane^11^,^12^. It was also demonstrated that IL-1 could induce natriuresis in the rat model^13^. Using cultures of inner medullary, cortical collecting duct and proximal tubular renal cells *in vitro*, the potential mechanisms were found to involve a reduction in the Na/K ATPase function mediated by prostaglandin E2^14^,^15^,^16^,^17^,^18^ and by increasing tissue levels of nitric oxide, which is a potent suppressor of the epithelial Na/K ATPase by the intracellular messenger cGMP and through protein kinase G modification^19^,^20^. An activation of the autoimmune system in SLE can cause CD8 + T-cells and macrophages to produce pro-inflammatory cytokines and chemokines, such as interleukin (IL)-1, IL-6, IL-8 and TNF-α^21^,^22^,^23^,^24^.

Because the degree of proteinuria did not differ in SLE children with hyponatremia than those without and our children with SLE did not have nephrotic syndrome or severe renal disease, hypoalbuminemia might occur by severe inflammation through a disease activity of SLE. Hypoalbuminemia has been regarded as an important cause of appreciable hypovolemic hyponatremia^25^. Also, it was thought that decreased serum calcium levels could be caused by hypoalbuminemia in our children’s cohort.

We found that hyponatremia was associated with increased ESR, CRP, and SLEDAI and decreased serum albumin and C3 levels by various statistical methods (univariate, multivariate and correlation analyses) in our cohorts of SLE, suggesting that hyponatremia in SLE is closely related to more severe inflammation. Although not studied yet in SLE, there have been some evidences and our hypothetical background showing that IL-1β or IL-6 might increase antidiuretic hormone (ADH) secretion, leading to hyponatremia^26^,^27^,^28^,^29^,^30^,^31^. Mastorakos *et al.* reported that plasma ADH levels were increased after injection of IL-6 in cancer patients, suggesting that IL-6 activated the magnocellular ADH-secreting neurons, which could be involved in the development of SIADH^26^. Ohta *et al.* performed animal experiments and intravenous administrations of IL-1β increased ADH^27^. Because IL-6 and IL-1β are all important cytokines in the pathogenesis of SLE both in animal models and human SLE^21^,^22^,^23^,^24^, there is a possibility that increased these cytokines could be implicated in the pathogenesis of hyponatremia in the patients with SLE. To validate this hypothesis, we analyzed the relationship between serum sodium and IL-6 levels in an independent cohort of adults with SLE and found that serum sodium levels were correlated negatively with serum IL-6 levels (r = −0.317, p = 0.003), which was more prominent in SLE patients who were not treated with steroids (r = −0.424, p = 0.017). In SLE patients who were not treated with steroids, serum sodium levels were correlated negatively with urine specific gravity which reflect the effect of ADH with a borderline significance (r = −0.326, p = 0.073).

Our study has some limitations, such as small sample size due to the rarity of lupus in Korea, retrospective study design. Nevertheless, the current data are noteworthy because we firstly showed that hyponatremia reflected disease activity of SLE in two independent cohorts of children and adults with SLE. Therefore, clinicians should recognize this electrolyte disturbance in SLE and pediatricians should pay more attention to the risk for exacerbating hyponatremia by administering a hypotonic fluid in children. Further studies are necessary to evaluate the exact molecular mechanism of hyponatremia in SLE and to elucidate whether our findings are also relevant in a large cohort of SLE in the future.

## Patients and Methods

### A cohort of children and adolescents with SLE

We retrospectively analyzed the data of 37 children (male:female = 4:33) who had been diagnosed with SLE in Severance Children’s Hospital for 20 years from the years 1991 to 2010. Laboratory examinations were collected two times at the stage of disease activity and at follow-up after 22.2 ± 28.3 months (range 1–123 months) in all patients. We divided the patients into two groups: group I (n = 11 samples, hyponatremia) and group II (n = 63 samples, no hyponatremia).

Medical charts were reviewed for clinical characteristics, such as age at onset, gender, and laboratory data, including WBC counts, hemoglobin, hematocrit, platelet counts, ESR, CRP, serum sodium, potassium, chloride, tCO_2_, calcium, phosphorus, BUN, creatinine, total protein, albumin, cholesterol, uric acid, AST, ALT, total bilirubin, and urinalysis.

Serum sodium assay was evaluated by using ion selective electrodes measurement by an automated chemistry analyzer (Olympus AU-2700, Beckman coulter, Mishima, Japan). Complete blood counts including platelet counts were analyzed by the Advia 2120i automated analyzer (Siemens Healthcare Diagnostics, Deerfield, IL, USA). CRP levels were measured by the latex-enhanced turbidimetric assay method using a Hitachi 7600 P module (Hitachi, Tokyo, Japan). ESR levels were measured by the TEST 1 (Alifax, Padova, Veneto, Italy). Serum C3 and C4 levels were measured by the automated Roche Diagnostics analyzer (Hitachi Cobas C501, Roche Diagnostics GmbH, Mannheim, Germany). Antinuclear antibodies and anti-ds DNA antibodies were detected by immunofluorescence using Crithidia luciliae (Department of Laboratory Medicine, Severance Hospital, Seoul). Strict quality control procedures were adopted.

### A cohort of adolescents and adults with SLE

Among 166 SLE patients who were followed up at Ajou University Hospital and in whom IL-6 levels were measured^23^, 84 were included in the study (age: mean 34.4 ± 12.1 years [range 16–69 years], male : female = 8:76) in whom serum sodium levels were available. Laboratory parameters of disease activity were recorded such as WBC counts, ESR, CRP, and urine specific gravity and SLEDAI was also calculated. Measurements of IL-2 and IL-6 in serum samples were performed by sandwich enzyme-linked immunosorbent assay (ELISA) using BD OptEIA sets (Pharmingen, San Diego, CA). All serum samples were measured in triplicate and diluted 1:1 in assay diluent for OptEIA ELISA sets. Briefly, after coating with primary anti-human IL-2 and IL-6 antibodies (Pharmingen) and blocking, 100 μg of diluted serum was loaded, and biotinylated secondary anti-human IL-2 and IL-6 monoclonal antibodies (Pharmingen) were added, respectively. The wells were incubated with streptavidine horseradish peroxidase conjugate, and colorimetric reaction was developed with 3,3′,5,5′-tetramethylbenzidine (TMB) substrate solution and terminated with 2 N H_2_SO_4_. Then, absorbance at 450 nm (reference, 570 nm) was read by an automated microplate reader (Benchmark, Bio-RAD, Hercules, CA). The serum cytokine levels were determined by comparison with a standard curve obtained using recombinant human IL-2 and IL-6, respectively^23^.

### Definitions

The diagnosis of SLE was based on the revised classification criteria of the American College of Rheumatology in 1997 in two cohorts of SLE^2^. The classification is based on 11 criteria and the diagnosis of SLE was made if any 4 or more of the 11 criteria are present, serially or simultaneously, during any interval of observation. The 11 criteria are as follows: malar rash, discoid rash, photosensitivity, oral ulcers, nonerosive arthritis involving 2 or more peripheral joints, pleuritis or pericarditis, renal disorder (persistent proteinuria >0.5 grams per day or >than 3+ if quantitation not performed or cellular casts–may be red cell, hemoglobin, granular, tubular, or mixed), neurologic disorder (seizures or psychosis), hematologic disorder (hemolytic anemia with reticulocytosis or leukopenia <4,000/mm^3^ on ≥2 occasions or lymphopenia <1,500/mm^3^ on ≥2 occasions or thrombocytopenia <100,000/mm^3^ in the absence of offending drugs), immunologic disorder (anti-ds DNA in abnormal titer or anti-Sm or positive finding of antiphospholipid antibodies) and positive antinuclear antibody^2^.

Hyponatremia was defined as serum sodium level ≤135 mEq/L. SLE activity was considered serologically as the increased titers of anti-ds DNA antibodies and decreased complement levels, such as C3 < 90 mg/dL and C4 < 10 mg/dL and SLEDAI was also measured^1^.

### Statistical methods

Statistical analyses were performed, using the SPSS for Windows (SPSS Inc., Chicago, Illinois, USA) and MedCalc version 15.8 (MedCalc Software, Ostend, Belgium). The independent t-test and Mann-Whitney U test was used for continuous variables and expressed as mean ± standard deviation. Fisher’s exact test was used to analyze categorical variables. Correlation analysis was also carried out to determine the relationship between two variables by Spearman or Pearson correlation. We also analyzed whether the changes (Δ: follow-up levels- initial levels) of serum sodium correlated with the changes (Δ) of other parameters in a cohort of children with SLE. Multiple logistic regression analysis was used to find independent predictive factors for hyponatremia in lupus. To establish the predictive value of the parameters for predicting hyponatremia, ROC curves were plotted for laboratory tests. All differences were considered significant at a value of p < 0.05

This study design and the use of patients’ information stored in the hospital database were approved by the Institutional Review Board and the research ethics committee of Yonsei Severance Hospital and Ajou University Hospital. We were given exemption from getting informed consents by the two IRBs because the present study is a retrospective study and personal identifiers were completely removed and the data were analyzed anonymously. Our study was conducted according to the ethical standards laid down in the 1964 Declaration of Helsinki and its later amendments.

## Additional Information

**How to cite this article**: Shin, J. I. *et al.* Hyponatremia in patients with systemic lupus erythematosus. *Sci. Rep.* **6**, 25566; doi: 10.1038/srep25566 (2016).

## Supplementary Material

Supplementary Information

## Figures and Tables

**Table 1:** General laboratory findings of children with lupus with or without hyponatremia.

	Group I withhyponatremia (n = 11)	Group II withouthyponatremia (n = 63)	*P* value
WBC (/μL)	8,456 ± 6,081	6,831 ± 3,641	0.933
ESR (mm/hr)	48.8 ± 31.9	29.9 ± 23.3	0.073
CRP (mg/dL)	3.6 ± 6.8	0.6 ± 0.8	0.478
Hb (g/dL)	10.8 ± ± 2.2	11.2 ± 2.1	0.140
Hct (%)	31.5 ± 6.6	32.7 ± 6.5	0.138
PLT (/μL)	226,364 ± 145,007	247,694 ± 107,885	0.418
Na (mmol/L)	131.4 ± 4.1	139.1 ± 2.3	<0.001
K (mmol/L)	3.7 ± 0.6	4.1 ± 0.5	0.105
Cl (mmol/L)	98.4 ± 9.3	104.5 ± 3.3	0.004
tCO_2_ (mmol/L)	22.7 ± 5.1	23.0 ± 3.1	0.994
BUN (mg/dL)	12.8 ± 4.5	11.7 ± 4.9	0.456
Creatinine (mg/dL)	0.6 ± 0.1	0.6 ± 0.2	0.994
AST (IU/L)	73 ± 70	33 ± 38	0.004
ALT (IU/L)	58 ± 79	24 ± 22	0.074
Total protein (g/dL)	6.7 ± 1.3	6.8 ± 0.9	0.330
Albumin (g/dL)	3.3 ± 0.4	4.0 ± 0.8	0.002
Uric acid (mg/dL)	5.0 ± 1.6	4.8 ± 1.2	0.835
Cholesterol (mg/dL)	150 ± 49	177 ± 83	0.176
Calcium (mg/dL)	8.0 ± 1.1	9.0 ± 0.8	0.004
Phosphorus (mg/dL)	3.3 ± 1.4	4.1 ± 1.0	0.124
Total bilirubin (mg/dL)	0.8 ± 0.8	0.6 ± 0.8	0.184
SLEDAI (score)	6.9 ± 6.1	3.1 ± 3.4	0.026

*WBC* white blood cell, *ESR* erythrocyte sedimentation rate, *CRP* C-reactive protein, *Hb* hemoglobin, *Hct* hematocrit, *PLT* platelet, *Na* Sodium, *K* potassium, *Cl* Chloride, *tCO*_*2*_ total carbon dioxide, *BUN* blood urea nitrogen, *AST* aspartate aminotransferase, *ALT* alanine aminotransferase, *SLEDAI* Systemic lupus erythematosus disease activity index.

**Table 2:** Immunologic laboratory findings and proteinuria of children with lupus with or without hyponatremia.

	Group I (n = 11)	Group II (n = 63)	*P* value
C3 (mg/dL)	38.4 ± 26.1	67.3 ± 28.6	0.004
C4 (mg/dL)	8.2 ± 6.5	13.8 ± 8.8	0.028
ANA	10(90.9%)	51(81.0%)	0.676
anti ds-DNA	8(72.7%)	29(46.0%)	0.190
Proteinuria	3(27.3%)	14(22.2%)	0.707
Anti-Sm	1/2(50%)	7/16(43.8%)	1.000
Anti-Ro	3/7(42.9%)	9/30(30%)	0.659
Anti-La	0/7(0%)	4/29(13.8%)	0.566
Anti-RNP	1/1(100%)	5/8(62.5%)	1.000
Anti-platelet antibody	2/4(50%)	2/4(50%)	–
LA	1/1(100%)	2/3(66.7%)	1.000
ACL	1/2(50%)	1/8(12.5%)	0.378
Anti-β2 GPI	0/5(0%)	0/5(0%)	–

*C3* complement component 3, *ANA* antinuclear antibody, *Anti-ds DNA* Anti**-**double-stranded DNA (Deoxyribonucleic acid), *Anti-RNP* Anti-Ribonucleoprotein, *LA* Lupus anticoagulant, *ACL* anticardiolipin antibodies, *Anti-β2 GPI* Anti-β2-glycoprotein I.

**Table 3:** Multiple logistic regression analysis of laboratory parameters associated with the development of lupus-associated hyponatremia in children with lupus.

	Odds ratio	95% CI	*P* value
ESR (mm/hr)	1.066	1.006–1.130	0.03
Chloride (mmol/L)	2.054	1.229–3.433	0.006
C3 (mg/dL)	1.069	1.006–1.136	0.031

*ESR* erythrocyte sedimentation rate, *C3* complement component 3.

**Table 4:** Correlations among key laboratory findings in children with lupus.

	Na	C3	ESR	Cl	Ca	Alb	AST	SLEDAI
Na	.	0.008	0.001(−)	0.001	<0.0001	<0.0001	0.004(−)	0.060
C3	0.008	.	0.003(−)	0.025	<0.0001	<0.0001	0.001(−)	<0.0001(−)
ESR	0.001(−)	0.003(−)	.	0.545	0.004(−)	0.001(−)	0.107	0.015
Cl	0.001	0.025	0.545	.	0.375	0.157	0.034(−)	0.612
Ca	<0.0001	<0.0001	0.004(−)	0.375	.	<0.0001	0.004 (−)	0.058
Alb	<0.0001	<0.0001	0.001(−)	0.157	<0.0001	.	<0.0001(−)	0.016(−)
AST	0.004(-)	0.001(−)	0.107	0.034(−)	0.004(−)	<0.0001(−)	.	0.002
SLE DAI	0.060	<0.0001(−)	0.015	0.612	0.058	0.016(−)	0.002	.

*Na* Sodium*, C3* complement component 3, *ESR* erythrocyte sedimentation rate, *Cl* Chloride, *Ca* Calcium, *Alb* Albumin, *AST* aspartate aminotransferase, *SLEDAI* Systemic lupus erythematosus disease activity index, (−): negative correlation.

**Table 5:** Correlations among changes in laboratory findings in children with lupus.

	ΔNa	ΔC3	ΔC4	ΔHb	ΔHct	ΔCl	ΔCa	ΔP	ΔAlb	ΔSLEDAI
ΔNa	.	0.003	0.030	0.039	0.009	0.004	0.019	0.017	0.003	0.313
ΔC3	0.003	.	<0.0001	<0.0001	0.003	0.706	0.001	0.025	<0.0001	0.052(−)
ΔC4	0.030	<0.0001	.	0.001	0.013	0.840	0.060	0.048	0.002	0.044(−)
ΔHb	0.039	<0.0001	0.001	.	<0.0001	0.739	0.028	0.197	0.037	0.006(−)
ΔHct	0.009	0.003	0.013	<0.0001	.	0.473	0.040	0.176	0.058	0.008(−)
ΔCl	0.004	0.706	0.840	0.739	0.473	.	0.564	0.949	0.418	0.208
ΔCa	0.019	0.001	0.060	0.028	0.040	0.564	.	0.001	<0.0001	0.391
ΔP	0.017	0.025	0.048	0.197	0.176	0.949	0.001	.	0.013	0.712
ΔAlb	0.003	<0.0001	0.002	0.037	0.058	0.418	<0.0001	0.013	.	0.170
ΔSLEDAI	0.313	0.052(−)	0.044(−)	0.006(−)	0.008(−)	0.208	0.391	0.712	0.170	.

*Na* Sodium*, C3* complement component 3, *C4* complement component 4, *Hb* Hemoglobin, *Hct* Hematocrit*, Cl* Chloride, *Ca* Calcium, *P* Phosphorus, *Alb* Albumin, SLEDAI Systemic lupus erythematosus disease activity index, (−): negative correlation.

**Table 6:** Correlations among key laboratory findings in adults with lupus.

	Na	WBC	ESR	CRP	SLE DA1	IL-6	IL-2	Steroid	SG
Na	.	0.373	0.070	0.446	0.716	0.003(−)	0.389	0.710	0.486
WBC	0.373	.	0.623	0.232	0.256	0.399	0.487	0.005	0.865
ESR	0.070	0.623	.	0.026	0.438	0.010	0.587	0.915	0.293
CRP	0.446	0.232	0.026	.	0.092	0.004	1.000	0.424	0.012(−)
SLEDAI	0.716	0.256	0.438	0.092	.	0.051	0.321	0.305	0.801
IL-6	0.003(−)	0.399	0.010	0.004	0.051	.	0.339	0.918	0.798
IL-2	0.389	0.487	0.587	1.000	0.321	0.339	.	0.979	0.032
Steroid	0.710	0.005	0.915	0.424	0.305	0.918	0.979	.	0.644
SG	0.486	0.865	0.293	0.012(−)	0.801	0.798	0.032	0.644	

*Na* Sodium*, WBC* white blood cell, *ESR* erythrocyte sedimentation rate, *CRP* C-reactive protein*, SLEDAI* Systemic lupus erythematosus disease activity index*, IL-6* Interleukin-6, *IL-2* Interleukin-2, *SG* urine specific gravity, (−): negative correlation.
