# Supplementary material for: Hyponatremia in patients with systemic lupus erythematosus
Source: Sci Rep. 2016 May 19;6:25566. doi: 10.1038/srep25566 (PMC4872139; doi:10.1038/srep25566)

**Supplementary tables**

**Hyponatremia in patients with systemic lupus erythematosus**

Jae Il Shin^1, 2*^, Se Jin Park^3*^, Chang-Hee Suh^4*^, Geum Hwa Lee^1^, Min Woo Hur^1^,

Song Yi Han^1^, Dong Soo Kim^1^ & Ji Hong Kim^1, 5^

1. Department of Pediatrics, Yonsei University College of Medicine, Seoul, Korea
2. Department of Pediatric Nephrology, Severance Children’s Hospital, Seoul, Korea
3. Department of Pediatrics, Ajou University School of Medicine, Daewoo General Hospital, Geoje, Korea
4. Department of Rheumatology, Ajou University School of Medicine, Suwon, Korea
5. Department of Pediatrics, Gangnam Severance Hospital, Yonsei University College of Medicine, Seoul, Korea

* Jae Il Shin, Se Jin Park, and Chang-Hee Suh contributed equally to this work.

**Supplementary table S1. Demographic and laboratory findings of adults with lupus with (Group 1) or without hyponatremia (Group 2)**

**Supplementary table S2. Correlations among key laboratory findings in adults with lupus who were treated with steroids**

**Supplementary table S3. Correlations among key laboratory findings in adults with lupus who were not treated with steroids**

**Supplementary table S4. Demographic and laboratory findings of combined cohorts with lupus with (Group 1) or without hyponatremia (Group 2)**

**Supplementary table S5. Multiple logistic regression analysis of parameters associated with the development of lupus-associated hyponatremia in combined cohorts with lupus**

**Supplementary table S6. Correlations among key laboratory findings in combined cohorts with lupus**

**Supplementary table S7.** **Receiver operating characteristic curve of various parameters for predicting hyponatremia in patients with lupus.**

**Supplementary figure S1. Receiver operating characteristic curve of various parameters for predicting hyponatremia in patients with lupus.**

**Supplementary table S1. Demographic and laboratory findings of adults with lupus with (Group 1) or without hyponatremia (Group 2)**

|  | Group 1 (n= 7) | Group 2 (n=77) | *P*-value |
| --- | --- | --- | --- |
| Age (years) | 32.0 ± 14.5 | 34.6 ± 11.9 | 0.512 |
| Na (mmol/L) | 134.9 ± 0.4 | 140.4 ± 2.7 | <0.0001 |
| WBC (/μL) | 6,912 ± 5,964 | 5,964 ± 3,090 | 0.254 |
| ESR (mm/hr) | 30.4 ± 15.2 | 29.6 ± 25.5 | 0.388 |
| CRP (mg/dL) | 2.1 ± 4.3 | 0.3 ± 0.4 | 0.077 |
| SLEDAI(score) | 5.6 ± 5.6 | 8.6 ± 6.7 | 0.283 |
| IL-6 (pg/mL) | 24.3 ± 53.1 | 3.7 ± 7.2 | 0.312 |
| IL-2 (pg/mL) | 5.4 ± 5.1 | 7.5 ± 14.9 | 0.627 |
| The dose of steroid (mg/day) | 20.4 ± 23.6 | 12.8 ± 15.1 | 0.583 |
| Urine specific gravity | 1.02 ± 0.004 | 1.02 ± 0.006 | 0.650 |

*Na* Sodium, *WBC* white blood cell, *ESR* erythrocyte sedimentation rate, *CRP* C-reactive protein, *SLEDAI* Systemic lupus erythematosus disease activity index*, IL-6* Interleukin-6, *IL-2* Interleukin-2,

**Supplementary table S2. Correlations among key laboratory findings in adults with lupus who were treated with steroids**

|  | Na | WBC | ESR | CRP | SLEDAI | IL-6 | IL-2 | Steroid | SG |
| --- | --- | --- | --- | --- | --- | --- | --- | --- | --- |
| Na | . | 0.391 | 0.015(-) | 0.267 | 0.761 | 0.075 | 0.857 | 0.067 | 0.756 |
| WBC | 0.391 | . | 0.517 | 0.086 | 0.964 | 0.515 | 0.769 | 0.357 | 0.362 |
| ESR | 0.015(-) | 0.517 | . | 0.095 | 0.886 | 0.186 | 0.043(-) | 0.353 | 0.281 |
| CRP | 0.267 | 0.086 | 0.095 | . | 0.440 | 0.059 | 0.259 | 0.871 | 0.005(-) |
| SLEDAI | 0.716 | 0.964 | 0.886 | 0.440 | . | 0.537 | 0.594 | 0.055 | 0.271 |
| IL-6 | 0.075 | 0.515 | 0.186 | 0.059 | 0.537 | . | 0.730 | 0.676 | 0.353 |
| IL-2 | 0.857 | 0.769 | 0.043 | 0.259 | 0.594 | 0.730 | . | 0.662 | 0.280 |
| Steroid | 0.067 | 0.357 | 0.353 | 0.871 | 0.055 | 0.676 | 0.662 | . | 0.254 |
| SG | 0.756 | 0.362 | 0.281 | 0.005(-) | 0.271 | 0.353 | 0.280 | 0.254 |  |

*Na* Sodium*, WBC* white blood cell, *ESR* erythrocyte sedimentation rate, *CRP* C-reactive protein*, SLEDAI* Systemic lupus erythematosus disease activity index*, IL-6* Interleukin-6, *IL-2* Interleukin-2, *Steroid* the dose of steroids, *SG* urine specific gravity

(-): negative correlation

**Supplementary table S3. Correlations among key laboratory findings in adults with lupus who were not treated with steroids**

|  | Na | WBC | ESR | CRP | SLEDAI | IL-6 | IL-2 | SG |
| --- | --- | --- | --- | --- | --- | --- | --- | --- |
| Na | . | 0.623 | 0.868 | 0.736 | 0.381 | 0.017(-) | 0.122 | 0.073(-) |
| WBC | 0.623 | . | 0.984 | 0.959 | 0.048(-) | 0.653 | 0.262 | 0.962 |
| ESR | 0.868 | 0.984 | . | 0.138 | 0.274 | 0.005 | 0.227 | 0.651 |
| CRP | 0.736 | 0.959 | 0.138 | . | 0.079 | 0.009 | 0.190 | 0.990 |
| SLEDAI | 0.381 | 0.048(-) | 0.274 | 0.079 | . | 0.032 | 0.449 | 0.383 |
| IL-6 | 0.017 | 0.653 | 0.005 | 0.009 | 0.032 | . | 0.291 | 0.391 |
| IL-2 | 0.122 | 0.262 | 0.227 | 0.190 | 0.449 | 0.291 | . | 0.052 |
| SG | 0.073(-) | 0.962 | 0.651 | 0.990 | 0.383 | 0.391 | 0.052 |  |

*Na* Sodium*, WBC* white blood cell, *ESR* erythrocyte sedimentation rate, *CRP* C-reactive protein*, SLEDAI* Systemic lupus erythematosus disease activity index*, IL-6* Interleukin-6, *IL-2* Interleukin-2, *SG* urine specific gravity

(-): negative correlation

**Supplementary table S4. Demographic and laboratory findings of combined cohorts with lupus with (Group 1) or without hyponatremia (Group 2)**

|  | Group 1 (n= 18) | | Group 2 (n=140) | *P*-value |
| --- | --- | --- | --- | --- |
| WBC (/μL) | | 7,856 ± 4,959 | 6,354 ± 3,365 | 0.355 |
| ESR (mm/hr) | | 41.2 ± 27.3 | 29.7 ± 24.6 | 0.053 |
| CRP (mg/dL) | | 3.0 ± 5.8 | 0.4 ± 0.6 | 0.053 |
| SLEDAI | | 6.3 ± 5.7 | 6.3 ± 6.1 | 0.815 |

*WBC* white blood cell, *ESR* erythrocyte sedimentation rate, *CRP* C-reactive protein, *SLEDAI* Systemic lupus erythematosus disease activity index

**Supplementary table S5. Multiple logistic regression analysis of parameters associated with the development of lupus-associated hyponatremia in combined cohorts with lupus**

|  | **Odds ratio** | **95% CI** | ***P* value** |
| --- | --- | --- | --- |
| WBC | 1.000 | 1.000-1.000 | 0.605 |
| ESR | 0.983 | 0.962-1.004 | 0.103 |
| CRP | 1.480 | 1.055-2.075 | 0.023 |
| SLEDAI | 0.983 | 0.909-1.109 | 0.938 |

*WBC* white blood cell, *ESR* erythrocyte sedimentation rate, *CRP* C-reactive protein, *SLEDAI* Systemic lupus erythematosus disease activity index

**Supplementary table S6. Correlations among key laboratory findings in combined cohorts with lupus**

|  | Na | SLEDAI | CRP | ESR | WBC |
| --- | --- | --- | --- | --- | --- |
| Na | . | 0.886 | <0.0001(-) | 0.023(-) | 0.374 |
| SLEDAI | 0.886 | . | 0.523 | 0.023 | 0.008(-) |
| CRP | <0.0001(-) | 0.523 | . | 0.209 | <0.0001 |
| ESR | 0.023(-) | 0.023 | 0.209 | . | 0.982 |
| WBC | 0.374 | 0.008(-) | <0.0001 | 0.982 | . |

*Na* Sodium, *SLEDAI* Systemic lupus erythematosus disease activity index*, CRP* C-reactive protein, *ESR* erythrocyte sedimentation rate, *WBC* White blood cell

**Supplementary table S7. Receiver operating characteristic curve of various parameters for predicting hyponatremia in patients with lupus.**

| **Variables** | **Area under curve** | **Standard error** | ***P* value** | **95% CI** |
| --- | --- | --- | --- | --- |
| SLEDAI (children and adults) | 0.743 | 0.082 | 0.029 | 0.583-0.904 |
| ESR (children and adults) | 0.768 | 0.086 | 0.016 | 0.600-0.936 |
| AST (children) | 0.770 | 0.091 | 0.015 | 0.592-0.947 |
| C3 (children) | 0.746 | 0.100 | 0.027 | 0.551-0.941 |
| Albumin (children) | 0.777 | 0.066 | 0.013 | 0.648-0.906 |

*SLEDAI* Systemic lupus erythematosus disease activity index*, ESR* erythrocyte sedimentation rate, *AST* aspartate aminotransferase*, C3* complement component 3

**Supplementary figure S1. Receiver operating characteristic curve of various parameters for predicting hyponatremia in patients with lupus.**


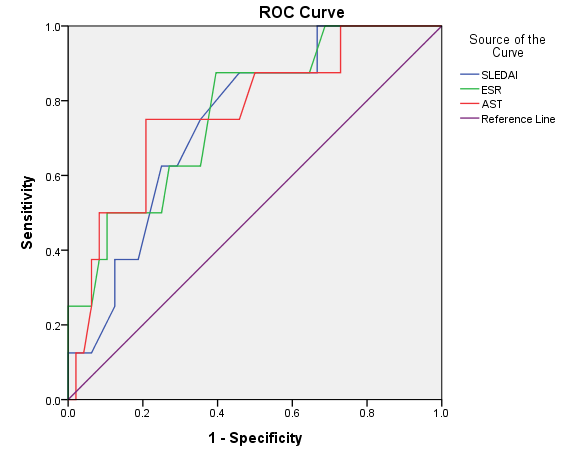


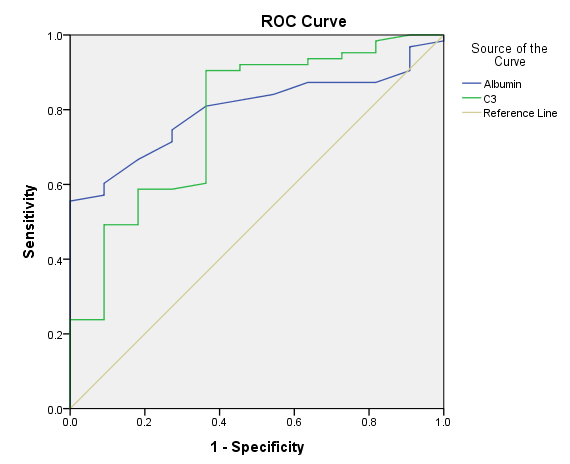

Supplement: Supplementary Information [file srep25566-s1.doc]
